# Recent advances in hepatic encephalopathy

**DOI:** 10.12688/f1000research.11938.1

**Published:** 2017-09-04

**Authors:** Victoria Liere, Gurkarminder Sandhu, Sharon DeMorrow

**Affiliations:** 1 Department of Medical Physiology, College of Medicine, Texas A&M Health Science Center, Temple, TX, USA; 2 Department of Internal Medicine, Baylor Scott & White, Temple, TX, USA; 3 Central Texas Veterans Healthcare System, Temple, TX, USA

**Keywords:** Hepatic encephalopathy, acute liver failure, chronic liver injury, neuroinflammation, pathogenesis

## Abstract

Hepatic encephalopathy describes the array of neurological alterations that occur during acute liver failure or chronic liver injury. While key players in the pathogenesis of hepatic encephalopathy, such as increases in brain ammonia, alterations in neurosteroid levels, and neuroinflammation, have been identified, there is still a paucity in our knowledge of the precise pathogenic mechanism. This review gives a brief overview of our understanding of the pathogenesis of hepatic encephalopathy and then summarizes the significant recent advances made in clinical and basic research contributing to our understanding, diagnosis, and possible treatment of hepatic encephalopathy. A literature search using the PubMed database was conducted in May 2017 using “hepatic encephalopathy” as a keyword, and selected manuscripts were limited to those research articles published since May 2014. While the authors acknowledge that many significant advances have been made in the understanding of hepatic encephalopathy prior to May 2014, we have limited the scope of this review to the previous three years only.

## Introduction

Advanced liver disease is well known for its systemic consequences, notably its profound effects on brain function, in its most classic form, known as hepatic encephalopathy (HE). HE is currently defined as a brain dysfunction secondary to liver insufficiency and/or portosystemic shunting that manifests as a broad spectrum of neuropsychiatric abnormalities ranging from subclinical alterations to coma ^1^. It remains a diagnosis of exclusion ^1^. HE is frequent: overt HE is found in some 30–40% of patients with cirrhosis ^1^. In 2009, HE resulted in 22,931 hospitalizations with an average cost of each stay ranging from $46,663–$63,108 ^2^. This highlights both the frequency of HE and the economic burden it currently places on the US.

The broad spectrum of HE has led to the development of multiple grading and classification systems to better categorize the severity of disease. It is currently a recommendation by the American Association for the Study of Liver Disease (AASLD) that HE should be classified according to the type of underlying disease, severity of manifestations, and precipitating factors ^1^. Types A, B, and C of HE define the underlying disease. Type A (acute) is due to acute liver failure (ALF), type B (bypass) is due to portosystemic shunting without intrinsic liver disease, and type C (cirrhosis) is due to cirrhosis ^1^. Each type has differing symptoms at presentation owing to the varying etiologies.

ALF, with the original term being fulminant hepatic failure, is defined as severe liver injury in the absence of pre-existing liver disease. The etiology of ALF is complex, ranging from viruses and drugs to genetic causes, with a vast majority remaining idiopathic ^3^. In developing nations, hepatitis (A, B, and C) is the most common cause of ALF, while in the US, acetaminophen toxicity accounts for 39% of ALF cases ^3^. Physical exam findings are often non-specific and generally reflect severe underlying liver dysfunction or complications thereof. ALF is currently a clinical diagnosis, made in the setting of acute liver injury in the context of abnormal liver tests (including dramatically elevated aminotransferases, and most importantly evidence of hepatocyte dysfunction demonstrated by a prolonged PT and increased INR ^3^). Unique to type A is its association with increased intracranial pressure (ICP), thus carrying a risk for cerebral herniation ^1^. It is believed that increased ICP results from cerebral edema secondary to hyperammonemia ^3^. One current proposed mechanism is that in ALF the development of hyperammonemia outpaces the compensatory mechanisms seen in chronic liver disease ^3^. Subsequently, ammonia increases intracellular osmolarity when it is metabolized to glutamine, resulting in cerebral edema causing increased ICP ^3^.

Type B HE is secondary to portosystemic bypass or bypass with no intrinsic hepatocellular disease. Precipitating factors of HE in type B are factors that are known to increase ammonia, such as azotemia, infection, GI bleed, lactulose noncompliance, and constipation ^4^. The most serious complication of transjugular intrahepatic portosystemic shunts (TIPS) is chronic recurrent HE that is refractory to standard treatment ^4^. If severe enough, shunt revision may be warranted ^4^.

Type C HE is associated with liver cirrhosis accompanied with either portal hypertension or portosystemic shunts ^1^. The causes of cirrhosis are vast, ranging from viral, autoimmune, chronic biliary disease, and fatty liver diseases to rare storage diseases such as hemochromatosis and Wilson’s disease ^5^. Signs and symptoms of cirrhosis are often present and include spider angioma, muscle wasting, jaundice, ascites, and gynecomastia and testicular atrophy in men. The most common form of HE in type C is minimal HE (MHE), which affects nearly 80% of cirrhotics ^6^. MHE affects the patient’s daily life, interfering with executive function including their working memory and orientation ^6^. Physical examination in MHE is often normal, and patients may present with subtle abnormalities that can be diagnosed by experts using specialized neurophysiologic tests, such as the critical flicker frequency test (CFF) ^6^. Currently, there is no gold standard test, and a number of studies are currently investigating the efficacy and use of them ^7^.

## Pathophysiology

As stated above, HE is broken into three major types because of its multiple etiologies. HE presents with a broad spectrum of symptoms ranging from subclinical to comatose. This wide range of presentation is due to the multifaceted pathophysiology that underlies this complex disease. Highlighted below is the role of ammonia and inflammation in the development of HE.

Currently, ammonia is the best-characterized neurotoxin in the pathogenesis of HE and also appears to be important in the genesis of astrocyte swelling ^8^. In healthy individuals, nitrogenous compounds, such as proteins, are metabolized by gut microflora and transported to the liver in the form of ammonia ^8^. In the liver, ammonia is metabolized by the urea cycle with the majority of the subsequent urea excreted renally ^8^. Advanced liver disease or portosystemic shunting leads to a buildup of ammonia in the blood ^8^. Ammonia that builds up in the blood is then able to cross the blood–brain barrier, where it is metabolized by astrocytes into glutamine ^8^. Hence, levels of glutamine start to accumulate and lead to astrocyte swelling. This swelling can trigger a downward spiral leading to increase in production of reactive oxygen and nitrogen species, which can downstream target gene transcription and translation ^9^. Animal models of HE emphasize that astrocyte and brain swelling is also a key feature. Among these features are swelling of astrocytes, vasoconstriction of blood vessels, increase in ICP, cerebral edema, reduced cerebral perfusion, and cerebral atrophy ^9^. There are conflicting studies regarding the correlation of levels of ammonia and the degree of encephalopathy; however, evidence suggests that a reduction in levels of ammonia leads to reduced brain swelling ^9^.

In addition to the ammonia hypothesis, inflammation and cytokines are thought to be major components in the development of HE, particularly in the setting of ALF with or without sepsis ^10^. Septic encephalopathy is a well-documented phenomenon that closely resembles HE ^10^. Although sepsis and ALF have varied pathogenic mechanisms, they share the same cardinal features of encephalopathy, cardiovascular collapse, and coagulopathy ^10^. Both sepsis and ALF result in an upregulation of inflammatory cytokines IL-1β, IL-6, and TNF-α ^10^. Studies have shown that these inflammatory cytokines compromise the blood–brain barrier and disrupt the permeability of the endothelial cells ^10^. It is not surprising that sepsis can precipitate and worsen HE ^10^. Cytokines, infection, and inflammation play a significant role in the development of HE. Studies have implicated both ammonia and inflammation in the pathogenesis of HE ^10^. Currently, it is proposed that these pathways have a synergistic effect on each other ^10^.

## Recent advances in understanding the pathogenesis of hepatic encephalopathy

**Figure 1.**
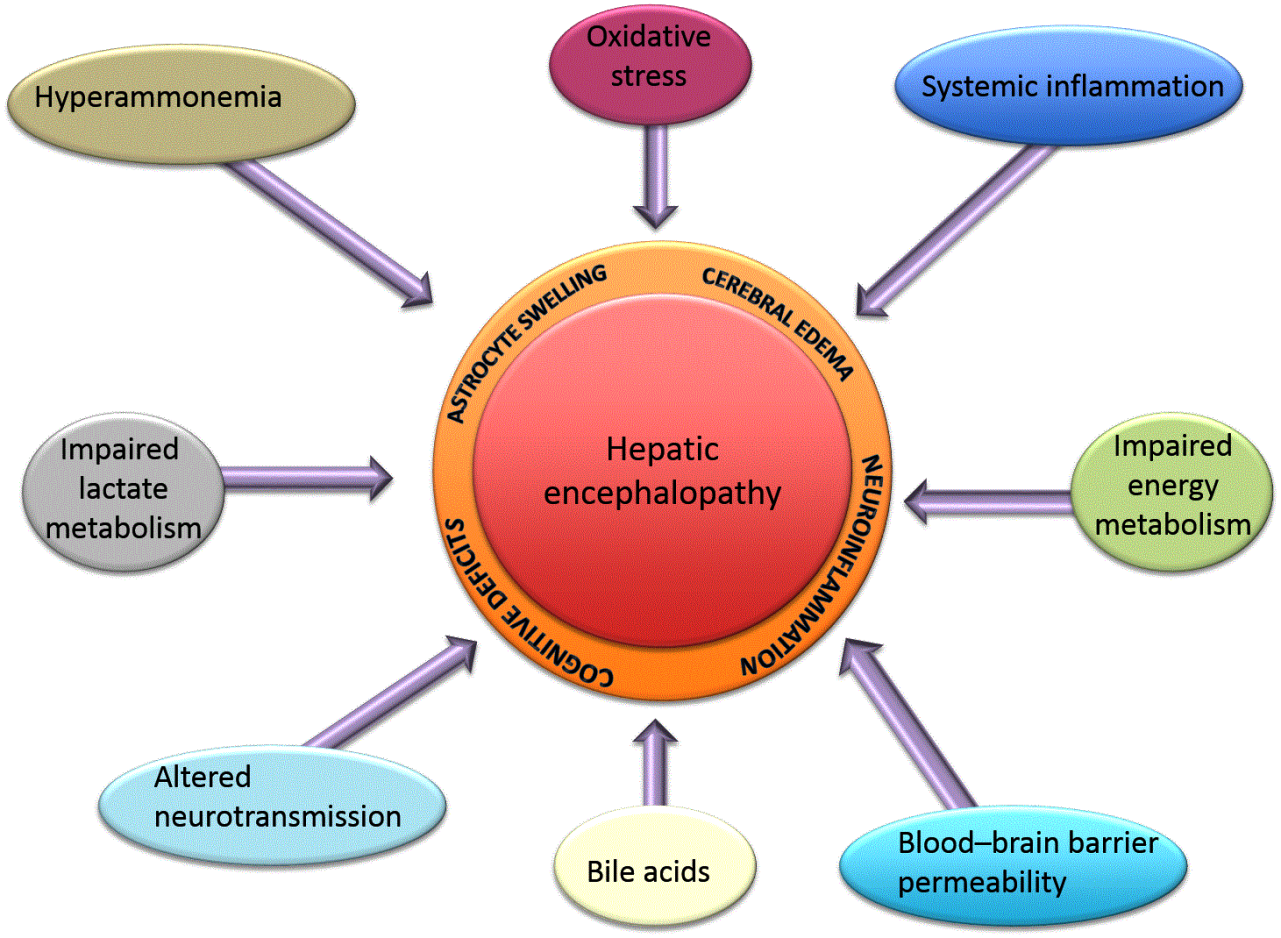
Summary of all factors known to contribute to the pathogenesis of hepatic encephalopathy.

Recent advances in the understanding of the pathogenesis of HE focus on the consequences of hyperammonemia and altered neurotransmission, and new studies have aimed to examine new potential targets such as bile acid signaling and their contribution to HE. Below is a summary of the latest findings (see also Figure 1).

### Hyperammonemia

A major contributing factor to the development of HE is a buildup of ammonia. Liver-specific glutamine synthetase knockout (a key enzyme in ammonia metabolism) led to dramatically increased systemic hyperammonemia and subsequent cerebral oxidative stress and cognitive changes ^11^, consistent with the concept that the liver is integral in the maintenance of ammonia homeostasis throughout the body.

In the brain under hyperammonemic conditions, astrocytes rapidly convert blood-derived ammonia into glutamine, increasing glutamine levels ^12^. An understanding of the full array of consequences of hyperammonemia on astrocytic function in the context of HE is, as yet, unappreciated. Historically, it was thought that the effects of increased cerebral ammonia were increased oxidative stress, osmotic pressure, and subsequent astrocyte swelling. However, recent studies have demonstrated that ammonia also has effects on many signal transduction pathways ^13– 15^, gene expression ^16^, and post-translational protein modifications ^17^, which together lead to subsequent impairment in astrocyte function and manifest as abnormal proliferation ^18, 19^, neurotransmitter release ^16^, and even cellular senescence ^18^. For example, hyperammonemia was recently associated with a transient increase in astrocyte intracellular calcium release ^13– 15^; the precise mechanism of action may be through activation of NMDA receptor ^13^, the transient receptor potential channel 1 ^14^, or the Cav1.2 L-type calcium channel ^15^, or, more likely, a combination of all of the above. Given that calcium-mediated effects are an early event of many different signal transduction pathways, the vast array of potential gene expression alterations due to hyperammonemia has not yet been defined.

A relatively novel mechanism by which ammonia may influence gene expression is via alterations in microRNA expression. MicroRNAs are small non-coding RNA sequences that play a role in gene silencing at the level of gene transcription as well as translation and can be regulated by oxidative stress. Exposure of astrocytes to ammonia dramatically altered microRNA profiles ^19^, leading to an alteration in many target genes, including heme oxygenase 1. Preventing ammonia-induced microRNA expression changes increased the expression of heme oxygenase 1 and prevented the ammonia-induced inhibition of astrocyte proliferation and prevented the associated cellular senescence ^19^.

Lastly, though not surprisingly, ammonia has been shown to alter the expression of genes involved in the regulation of astrocytic glutamate uptake and neurotransmitter release, such as the ephrin receptors and their ligands ^16^ and thrombospondin 1 ^20^. Specifically, the treatment of astrocytes in culture with ammonia increased ephrin receptor 4 via an as-yet-undefined pathway that includes glutamine synthetase, NADPH oxidase, and nitric oxide synthase activities ^16^. Conversely, chronic ammonia treatment decreased thrombospondin 1 expression in astrocytes, which subsequently decreased the expression of synaptic proteins in neurons ^20^.

Ammonia may also contribute to the cell-to-cell crosstalk that occurs in HE. Specifically, the treatment of brain endothelial cells with ammonia, a mixture of cytokines, or lipopolysaccharide appeared to release factors that contribute to astrocyte swelling via the activation of Toll-like receptor 4 ^21^. The understanding of cell–cell interactions and communication and the role of ammonia in HE is still quite limited. In further support of the notion that cell–cell communication is involved in ammonia-induced events during HE, strategies to uncouple the gap junctions in astrocyte cultures significantly attenuated key features of ammonia-induced neurotoxicity, such as membrane integrity, oxidative stress, and pro-inflammatory cytokine release ^22^.

### Oxidative stress

Oxidative stress and the generation of reactive oxygen and nitrogen species in the brain has long been associated with HE. It is thought that impaired mitochondrial function and downregulation of the expression of key anti-oxidation enzymes contribute to an increase in oxidative damage to membrane lipids, protein, and DNA ^23^.

Recent studies have emphasized the importance of oxidative damage in the pathogenesis of HE, with various experimental treatments aimed at reducing reactive oxygen/nitrogen species ^24– 31^ or restoring the activity of anti-oxidative enzymes such as catalase ^32^, superoxide dismutase ^32^, thioredoxin ^25^, or glutathione peroxidase ^25^. Of particular note was an elegant study by Bosoi *et al*. ^33^, in which the authors utilized a rodent model of hyperammonemia that was also treated with an inhibitor of glutathione; they found that a synergistic relationship between systemic oxidative stress and hyperammonemia was required for the development of brain edema in HE ^33^.

### Brain energy metabolism

The pathogenesis of HE has long been associated with impairment in cerebral energy metabolism with alterations in glucose utilization, glycolysis, and mitochondrial dysfunction (reviewed in Rama Rao and Norenberg ^34^). Of particular interest are the changes in cerebral lactate levels during HE. Specifically, increased blood and cerebral lactate levels have been demonstrated in patients with HE ^35^, in pig and rodent models of ALF ^36, 37^, and in acute hyperammonemia ^38^. However, the increase in cerebral lactate in models of type C HE is not as clear-cut. Increased lactate has been observed in the plasma of patients with cirrhosis compared to age- and sex-matched controls ^39^. Interestingly, there was no significant difference in plasma lactate levels between cirrhotic patients who exhibit symptoms of overt HE versus patients without overt HE, although the presence of MHE in the latter group could not be ruled out ^39^. A parallel change in lactate levels in the brain was not evident in a longitudinal study using 1H and 31P magnetic resonance spectroscopy in a rodent model of type C HE ^12^. Conversely, *de novo* synthesis of lactate was increased in the brain, and treatment with a lactate synthesis inhibitor attenuated HE-associated brain edema in this model ^40^. This disparity between these two opposing observations may lie in the methodology used to detect the differences. However, recently, it was demonstrated that the transport of lactate through connexin-containing hemichannels in astrocytes was impaired in the cerebral cortices of rats with type C HE owing, at least in part, to the actions of hyperammonemia ^41^. Given that the astrocyte–neuron lactate shuttle hypothesis suggests that lactate production in astrocytes is able to fuel and regulate neuronal activity ^42^, it was hypothesized that the impairment of lactate transport through hemichannels may be contributing to the pathogenesis of HE ^41^.

### Neuroinflammation

Brain inflammation (i.e. “neuroinflammation”) is a key feature in common with all types of HE and appears to be predominantly modulated by microglia, the resident macrophage-like cell in the brain. Indirect clinical evidence for microglial activation has been demonstrated by an upregulation of the microglial marker ionized calcium-binding adaptor molecule 1 (Iba-1), which was found to be increased in post mortem cortical brain tissue from patients with liver cirrhosis and HE compared to cirrhotic patients without HE ^43^. In addition, a comprehensive gene expression profile analysis showed that markers for both the pro-inflammatory M1 and the anti-inflammatory M2 microglial phenotypes were increased, suggesting that both groups can be found in patients with HE caused by cirrhosis ^44^.

The activation of microglia is a delicate balance between the pro-inflammatory and anti-inflammatory signals, which in physiological conditions favors the dampening of microglia activation ^45^. These signals may be derived from the microglia themselves or are as a result of cell-to-cell communication derived from neurons or astrocytes. Recently, the pro-inflammatory chemokine CCL2 was demonstrated to be increased in neurons in a mouse model of type A HE ^46^ and a concomitant decrease in the anti-inflammatory chemokine fractalkine ^45^, thereby dysregulating the balance between opposing pro- and anti-inflammatory signals acting on receptors on microglia resulting in microglia activation. Strategies to either block CCL2 receptors or increase fractalkine signaling inhibited the microglia activation and attenuated the cognitive dysfunction observed in this model of HE, although the precise mechanism by which the balance between these two opposing signals is dysregulated was not identified.

Interestingly, in the hyperammonemic rat model, microglia and astrocytes were activated with a concomitant increase in the expression of pro-inflammatory cytokines IL-1β and IL-6 ^47^, suggesting that ammonia alone is capable of inducing neuroinflammation during HE, although it is unlikely that the modulation of neuroinflammation is solely the consequence of hyperammonemia during HE.

Evidence to suggest a causal link between neuroinflammation and cognitive and motor function impairment during HE is mounting. Strategies that specifically target and dampen the neuroinflammatory signals also offer attenuation of cognitive and motor deficits ^46, 48– 52^, although care should be taken when interpreting data from experiments where the anti-inflammatory experimental agent is given systemically, as the mechanism of action may be via hepato-protection, thereby leading to a reduction in HE symptoms rather than as a direct modulatory effect on central neuroinflammation. While treatment strategies aimed at dampening systemic inflammation may be beneficial for both the underlying liver damage and the subsequent encephalopathy, from a basic science standpoint aimed at elucidating the pathogenic pathways associated with the development of HE, the distinction between the actions of an experimental compound on the brain versus its actions on the liver should be distinguished.

### Neurotransmitter dysfunction

The cognitive and neuromuscular deficits observed during HE are ultimately the result of altered neurotransmission, regardless of the mechanism ^53^. Interestingly, there are opposing effects on glutamatergic neurotransmission depending upon the type of HE with increased extracellular glutamate levels observed after ALF, and a dampened glutamatergic neurotransmission observed during chronic liver disease ^53^. During ALF, the activation of NMDA receptors on astrocytes downregulates the expression of Kir4.1, an inward rectifying potassium channel known to regulate ion and water homeostasis ^54^ and contribute to neuronal dysfunction in other neurodegenerative diseases ^55^. The precise role for Kir4.1 in type A HE is not clear. Furthermore, strategies to block NMDA receptors in rats with ALF reduced the HE-associated changes in cerebral blood flow and brain lactate as well as increased the kidney clearance of ammonia, which collectively delayed or prevented the HE-associated mortality ^56^. Conversely, during MHE due to chronic liver disease, memory impairment was associated with suppression of the glutamate–nitric oxide–cyclic guanosine monophosphate (GMP) pathway, which has been demonstrated in response to hyperammonemia ^57^ or increased intracranial dopamine ^58^, and strategies to restore this pathway also restore the learning ability of rats with HE ^57^.

Both type A and type C HE are associated with increased GABA-ergic tone. This is due to a number of factors: increased GABA concentrations ^59^, increased GABA receptor expression ^59^, and increased neurosteroids such as allopregnanolone, known to regulate GABA-ergic neurotransmission ^60^. Neurosteroids are steroids produced *de novo* in the brain ^61^. Their production and function are currently under investigation. Recently, experimental compounds aimed at either inhibiting neurosteroid synthesis ^62^ or inhibiting the enhanced activation of GABAA receptors by neurosteroids ^63^ attenuated the deficits in motor co-ordination, spatial memory, and circadian rhythms observed during HE ^62, 63^. These data were supported by the use of an inhibitor of another neurotransmitter system thought to be dysregulated during at least MHE: the dopamine system ^64^. However, how this system is altered during HE remains unclear. It has been hypothesized that an increase in dopamine release from cirrhotic liver into the bloodstream contributes to the pathogenic features of MHE by inhibiting the glutamate–nitric oxide–cyclic GMP pathway in the hippocampus, thereby altering learning and memory ability ^58, 64– 66^. Conversely, dopamine and serotonin levels were decreased in another model of MHE ^67^.

### Bile acids

Elevated circulating and cranial levels of bile acids have been observed in patients with HE as early as 1977, although our understanding of the implications of this observation are only just coming to light. Specifically, total bile acid content in the cerebrospinal fluid as well as in brain tissue was elevated in patients and rodent models of type A HE ^68, 69^ as well as type C HE ^70– 72^; however, the precise role of bile acids in HE remains controversial. Increased serum bile acids have been implicated in the increased blood–brain barrier permeability observed in a rat model of chronic liver disease ^70^, thereby allowing access of bile acids and other signaling molecules to the brain. In mice, azoxymethane (AOM) is used as a model for type A HE due to ALF ^69^. Indeed, in the AOM mouse model of type A HE, increased total bile acid content was observed in the frontal cortex, and strategies to reduce circulating bile acids (e.g. cholestyramine feeding or the use of a genetically modified mouse with impaired bile acid synthesis) proved neuroprotective ^69^.

Bile acids can exert their effects via many different receptors that result in an array of physiological responses. FXR is a nuclear bile acid receptor, and inhibition of FXR-mediated signaling in the frontal cortex conferred partial protection against the cognitive deficit that occurs during HE ^69^. Furthermore, aberrant bile acid signaling in the brain can also affect neuroinflammation by increasing pro-inflammatory CCL2 expression in neurons via a sphingosine-1-phosphate receptor 2-dependent mechanism, which subsequently leads to the activation of microglia and increase in pro-inflammatory cytokine expression ^73^. Conversely, TGR5 is a membrane-bound G-protein-coupled bile acid receptor, the expression of which has been shown to decrease in the brain in conditions of hyperammonemia ^74^. Activation of this receptor with specific agonists reduced the microglia activation and pro-inflammatory cytokine production observed in a mouse model of type A HE ^75^.

Many therapeutic strategies to treat HE, such as lactulose and metronidazole, abolish gut microbiota with the aim of reducing ammonia production while also influencing the conjugation of secondary bile acids. By altering the bile acid pool, they generate protective effects by altering bile acid signaling. In support of this notion, feeding mice a diet enriched in particular bile acids, which does not alter the total bile acid pool but alters the balance of individual bile acids, changed the susceptibility of mice to the development of HE ^69^. Specifically, mice fed a diet enriched with deoxycholic acid or cholic acid had significantly quicker neurological decline compared to control-fed or ursodeoxycholic acid-fed mice ^69^. These data raise the possibility that the elevation of cranial bile acids as well as a change in specific bile acids in the bile acid pool contribute to the neurological decline associated with HE; further clinical studies that manipulate bile acids are necessary to determine whether these effects will be recapitulated in patients with HE.

### Blood–brain barrier permeability

The role of blood–brain barrier permeability is controversial in HE. No robust evidence of its breakdown exists; however, it is highly likely that bile acids and pro-inflammatory cytokines may affect its permeability ^70, 76– 79^. In the AOM model of type A HE, permeability of the blood–brain barrier has been demonstrated as a late event during the development of HE ^78, 79^. Conversely, no changes in permeability could be detected by another group unless the mice were co-treated with trace amounts of lipopolysaccharide ^80^, suggesting a synergistic role for inflammation with other key HE-related features in the changes in blood–brain barrier permeability. Consistent with this notion was a study demonstrating that circulating transforming growth factor-β (TGFβ) may contribute to the increased permeability of the blood–brain barrier in ALF ^78^.

Evidence to suggest that blood–brain barrier permeability is increased in type C HE also exists. As stated above, increased permeability in the bile duct ligation model of HE is apparent and can be attributed, at least in part, to increased serum bile acids ^70^. Associated with this increased permeability was a decrease in expression of tight junction proteins ^76^.

## Recent advances in the diagnosis of hepatic encephalopathy

HE remains a diagnosis of exclusion with a broad spectrum of clinical manifestations. The multifaceted presentation poses many limitations to the diagnosis of HE. Guidelines by the AASLD emphasize the use of specialized psychometric tests in the diagnosis of HE ^81– 83^. A multitude of tests exist, with some of the most studied being the psychomotor hepatic encephalopathy score (PHES), CFF, Stroop effect test, and electroencephalogram (EEG) ^81, 84^.

PHES is a battery of pencil and paper tests aimed at diagnosing HE ^85^. PHES was studied in Turkey and Romania in order to standardize a normal value in a healthy population and also assess its utility ^85, 86^. Age and educational status were shown to affect score ^85^.

Apart from PHES, a newly developed electronic number connection test (eNCT) was investigated by Wuensh *et al*. ^87^. This test flashes the numbers 1–25 on a screen and requires the participant to click them in order while being timed; those patients with HE were found to be much slower ^87^. This test promises to be an easier and faster method than PHES ^87^.

In the CFF, patients are shown a light flicker that progressively decreases in frequency ^88^. They must identify the frequency at which the light flickers ^88^. CFF can be used as a measure of cortical function and the diagnosis of HE ^88^. Studies show that with a cut-off value of <39 Hz, CFF has a sensitivity of 39%, specificity of 82%, and diagnostic accuracy of 70.6% for MHE ^82, 88^. CFF is an important and simple tool for diagnosis and should be combined with the model for end stage liver disease (MELD) score for better diagnosis ^89^. MELD is a scoring system used to grade the severity of liver disease ^90^.

A prospective study of 117 consecutive patients with cirrhosis examined the administration of the CFF to detect HE ^91^. The authors found that MHE was associated with a reduced 5-year survival rate in patients with cirrhosis ^91^. CFF could be used in the future to improve the accuracy of prognosis in patients with cirrhosis ^91^.

Another study focused on Stroop testing, which tests psychomotor speed, attention, and cognitive flexibility ^92^ in the form of an app coined EncephalApp. The app’s reliability was tested compared to the gold standard tests and was validated by multicenter and multi-national analysis in the USA with over 800 subjects ^92^. Another validation of the EncephalApp was done with 437 cirrhotics and 308 controls, and it was compared with PHES and the inhibitory control test (ICT) ^93^. When compared against the controls, the app was found to be equivalent in predicting HE, thus proving to be a convenient and validated method for the diagnosis of HE ^93^.

Currently, the analysis of EEG patterns in children is used for grading HE in ALF ^94^. Nonetheless, EEG use in HE remains controversial. One study recorded EEGs in 69 healthy controls and 113 adults with cirrhosis ^95^. New spectral thresholds were calculated and optimized the performance of EEG for the diagnosis of HE ^95^. Though improvements were made in the use of EEG for the diagnosis of HE, validation is needed along with further research to understand the pathophysiological mechanism behind the EEG changes ^95– 97^.

Key changes in brain function have been studied with functional magnetic resonance imaging (MRI) ^98^ in combination with arterial-spin labeling to enhance the detection of HE ^99^. Connectivity status between 90 brain regions was assessed by measuring blood flow. HE showed impairment in the ganglia–thalamo–cortical circuits, with increased blood flow in the right putamen ^99^.

Acknowledging the multiple etiologies of HE, researchers investigated if the cause of HE affected its presentation on imaging. Neurocognitive, biochemical, and brain MRI changes have been examined in patients with cirrhotic HE and extrahepatic portal vein obstruction (EHPVO) HE ^100^. Serum studies showed no change in ammonia levels while cytokine levels were higher in cirrhotic HE ^100^. Cirrhotic HE showed imaging changes that were absent in EHPVO HE ^100^. Cirrhotic HE affected multiple brain sites ranging from the frontal lobe all the way to the brainstem ^100^.

Spontaneous brain activity can also be measured by examining the amplitude of low-frequency fluctuation (ALFF) in the MRI ^101^. It can be used as a biomarker for the detection of HE ^101^. Upcoming research focuses on ALFF in combination with blood oxygenation level-dependent functional MRI, creating new opportunities for the diagnosis of HE ^102^.

Cerebral blood flow is altered in HE. Regional cerebral blood flow was studied by comparing dogs with congenital portosystemic shunt with HE to controls using nuclear imaging ^103^. It showed decreased perfusion of subcortical and temporal regions in dogs with HE ^103^. This highlights the role that nuclear imaging, specifically single photon emission computed tomography, can play in helping diagnose HE ^103^.

Metabolic profiling analyzes body fluids or tissues by measuring low-molecular-weight compounds using either ^1^H-NMR spectroscopy or mass spectrometry techniques ^104^. Currently, it is used in hepatocellular carcinoma; however, its use in HE had not been previously studied ^104^. Studies have shown that metabolic profiling can distinguish overt HE, but it cannot discriminate between differing grades of HE or according to the severity of underlying liver disease ^104^.

## Recent research on the treatment of hepatic encephalopathy

### Gut microbiome

The gut microbiome is implicated in the pathogenesis of HE ^105– 108^. Urease-producing bacteria predominantly lead to elevated levels of ammonia and serve as a target for many treatments ^109^. A cross-sectional study set out to determine the species of gut microbiota predominant in HE and suggested that an absence of *Blastocystis* spp. led to dysbiosis.

Proton pump inhibitors (PPIs), a major treatment for gastroesophageal reflux, have been shown to be related to HE ^110– 112^. While the exact mechanism underlying this association is not known, it is currently thought to be due to alterations in the gut microbiome. PPIs have a known association with gut dysbiosis ^110– 112^. A case-control study of one million people in Taiwan followed for an average of nearly 14 years demonstrated that in patients with both cirrhosis and HE, 38% used a PPI ^112^; all categories of PPIs, except rabeprazole, led to an increased risk of HE ^112^. It is crucial to recognize the irreversible consequences that can occur with non-specific selection of treatment for reflux. Likewise, a meta-analysis showed that those with liver dysfunction who took a PPI had a higher risk of developing HE compared to those who did not take a PPI ^110^.

Recognizing the importance of the gut microbiome hypotheses has led to study of the role of probiotics in HE ^113, 114^. A meta-analysis of randomized trials suggested that probiotics are more effective in decreasing hospitalization rates, improving MHE, and preventing progression to overt HE ^115^. Similarly, a further meta-analysis by the Cochrane collaboration revealed that when compared to placebo, probiotics mildly improved recovery, decreased overt HE, improved quality of life, and mildly decreased plasma ammonia ^116– 118^. Of note, probiotics have no effect on mortality ^116– 118^. Because of the low quality of evidence, no direct conclusions can be made, as more research is needed ^116– 118^. A double-blind randomized placebo-controlled clinical trial studied the effectiveness of VSL#3 ^119^ in Chandigarh, India. This study demonstrated reductions in hospitalizations and severity of breakthrough episodes of HE as well as improvement in PHES and liver function in patients receiving VSL#3 ^119^.

Diet changes have been studied to determine their effects on cognitive function in patients with MHE with cirrhosis ^120^. One study in 2016 compared patients prescribed a diet with a caloric restriction of 30–35 kcal/kg and a protein intake of 1.0–1.5 g of vegetable protein/kg to a control group for six months ^120^. PHES was used to diagnose HE. They found that a higher proportion of patients on the prescribed diet had reversal of HE (71.1% versus 22.9%, *p*=0.001) along with a decreased incidence of overt HE in the treatment group (10% versus 21.7%, *p*=0.4) ^120^.

Alteration in the gut–brain axis in HE has led to investigations in determining the effectiveness of gut-selective antibiotics. Modulating the gut microbiota with the use of antibiotics such as rifaximin may improve cognitive performance in cirrhotics ^121– 126^. This hypothesis was tested in a study at the Hunter Holmes McGuire VA Medical Center ^127^. Patients with confirmed HE were prescribed rifaximin 550 mg oral twice a day over a period of eight weeks ^127^. Compared to the placebo group, the treatment group had cognitive improvement, decreased endotoxemia, and a modest decrease in bacteria of the small bowel ^127^.

A retrospective, observational, cross-sectional study examined the efficacy of different rifaximin dosing (400 mg three times daily versus 550 mg twice daily) ^128^. There was no difference in readmission rates between the two treatment groups ^128^. The long-term cost effectiveness of lactulose monotherapy versus lactulose plus rifaximin was studied in France ^129^. Despite having no effect on hospital length of stay, combination therapy led to lower readmission rates at 180 days compared to lactulose alone ^130^. The authors concluded that rifaximin was a cost-effective and affordable treatment for patients suffering from at least two prior events of HE ^129^.

### Hyperammonemia

As stated above, numerous studies have shown that ammonia is key in the pathogenesis of HE. Currently, the gold-standard treatment for the reduction of ammonia during HE is lactulose ^131^, although a number of other experimental drugs aimed at lowering ammonia have been trialed including ornithine phenylacetate (OP) ^9, 132– 134^ and L-ornithine L-aspartate (LOLA) ^135– 137^. A major breakthrough came with an investigation of bacterial-DNA translocation effects on hyperammonemia and neurocognitive scores in patients with HE after the use of lactulose ^131^. The authors showed that neurocognitive scores improved with lactulose use and also led to reduced bacterial-DNA translocation ^131^.

Evidence is mounting that the use of polyethylene glycol (PEG), with or without lactulose, is effective. A randomized controlled trial in 40 patients compared the use of lactulose versus combined lactulose and PEG in cirrhotic patients admitted with HE ^138^. There was a statistically significant decrease in length of hospital stay (in women) ^138^. Surprisingly, there was no effect on ammonia levels ^138^. Likewise, the HELP randomized trial found that, compared to lactulose, PEG led to more rapid HE resolution in the hospital and concluded that it was superior to standard lactulose therapy ^139^.

Studies are investigating albumin transfusion as a possible treatment for HE ^140^. A prospective randomized controlled trial investigated lactulose alone versus lactulose plus albumin ^140^. Combination treatment of lactulose plus albumin led to a decrease in levels of ammonia, IL-6, IL-18, and TNF-α and other endotoxins ^140^.

Studies are also investigating branched chain amino acid (BCAA) supplementation in advanced liver disease. The ratio of normal BCAAs to aromatic amino acids becomes reduced in HE ^141^. Randomized controlled studies show that BCAA supplementation does not prevent the development of HE but may slow down the progression of hepatic failure ^141, 142^. Despite BCAAs having no effect on mortality, 16 randomized controlled clinical trials have shown that BCAAs decrease the signs and symptoms of HE ^143^. Conflicting studies exist, with some stating BCAAs have no significant effect on HE ^144^.

Recent studies have examined the use of corticosteroid administration in patients with acute liver injury and ALF ^145^. A prospective observational study of 469 patients from 2004 to 2015 showed that high-dose therapy decreased the risk of HE, but the time to initiate therapy has yet to be established ^145^.

### Surgical treatment

As might be predicted, liver transplantation has been shown to improve the brain activity and cognitive function of patients with cirrhosis with or without HE ^146^. Zhang *et al*. studied the effect of liver transplant on resting-state brain activity by quantizing the ALFF before and one month after transplant. Cognitive function improved in both groups with and without encephalopathy, showing transplant as a viable treatment option for HE ^146^.

In a case report, a 73-year-old male presented with new-onset HE and was found to have a congenital portosystemic shunt with a normal liver histology. Correction of the shunt led to full reversal of his symptoms ^147^. This highlights a surgically correctable cause of acute HE without underlying liver disease.

An increase in shunt blood bypassing the liver owing to portal hypertension can result in HE. Surgical interventions to close these shunts have been studied as a possible treatment for HE ^148^. Percutaneous transhepatic obliteration (PTO) and percutaneous transhepatic sclerotherapy (PTS) are two methods used to close shunts in the setting of emergent variceal hemorrhage when other methods have failed ^148^. PTO is performed by placing metallic coils in the afferent veins to reduce blood flow, while PTS is performed by injecting a sclerosing agent ^148^. Both methods utilize an invasive transcutaneous intrahepatic puncture ^148^. A study of 37 patients with variceal hemorrhage and intractable HE underwent PTO/PTS ^148^. Blood ammonia levels greatly improved, dropping from 135 mg/dL to 65 mg/dL at six months, and there was also an improvement in HE ^148^.

TIPS is a procedure that places a stent between the hepatic vein and portal vein to alleviate portal hypertension ^149^. However, allowing blood to bypass the liver can worsen HE ^149, 150^. The size of the stent in TIPS has been associated with HE ^151, 152^. One study showed that the use of a larger diameter stent improved ascites without a significant change in HE ^151, 152^. Additionally, the consumption of a low-protein diet after TIPS can reduce the incidence of HE ^153^. Interestingly, antegrade embolization of spontaneous splenorenal shunt has also been employed to decrease the incidence of post-TIPS HE and has decreased the rate of variceal bleeding ^154^.

Balloon-occluded retrograde transvenous obliteration (BRTO) is an alternative or adjuvant to TIPS in the management of gastric varices ^155^. It works by abolishing gastrorenal or splenorenal shunts ^155^. A retrospective record-based study of patients who underwent BRTO for gastric variceal bleeding or refractory HE showed great improvement in grade of encephalopathy after BRTO ^155^.

## Conclusions

This review summarizes the advances made in basic and clinical research over the last three years that have furthered our understanding of the pathophysiology of HE and possible treatments. Recent developments in basic research have focused on the role of hyperammonemia, oxidative stress, neuroinflammation, neurotransmitter function, bile acids, and the blood–brain barrier. More research is needed in these areas to fully establish the mechanism behind these various components and the role they play in the pathophysiology of HE. The diagnosis of HE is being investigated and is expected to become more precise by optimizing and validating current scoring systems. It appears that at least some of these will be amenable to modern app technology. Research in non-invasive imaging is also active and promises to aid in the diagnosis of HE. Strides have been made in the treatment of HE by specifically targeting the gut microbiome and hyperammonemia.

## Abbreviations

AASLD, American Association for the Study of Liver Disease; ALF, acute liver failure; ALFF, amplitude of low-frequency fluctuation; AOM, azoxymethane; BCAA, branched chain amino acid; BRTO, balloon-occluded retrograde transvenous obliteration; CFF, critical flicker frequency test; EEG, electroencephalogram; EHPVO, extrahepatic portal vein obstruction; GMP, guanosine monophosphate; HE, hepatic encephalopathy; ICP, intracranial pressure; MELD, model for end stage liver disease; MHE, minimal hepatic encephalopathy; MRI, magnetic resonance imaging; PEG, polyethylene glycol; PHES, psychomotor hepatic encephalopathy score; PPI, proton pump inhibitor; PTO, percutaneous transhepatic obliteration; PTS, percutaneous transhepatic sclerotherapy; TIPS, transjugular intrahepatic portosystemic shunts.
